# Neutropenic Rat Thigh Infection Model for Evaluation of the Pharmacokinetics/Pharmacodynamics of Anti-Infectives

**DOI:** 10.1128/spectrum.00133-23

**Published:** 2023-06-01

**Authors:** Randhir Yedle, Mahesh Kumar Reniguntla, Ramesh Puttaswamy, Pradeep Puttarangappa, Somashekharayya Hiremath, Mahesh Nanjundappa, Ramesh Jayaraman

**Affiliations:** a TheraIndx Lifesciences Pvt. Ltd., Nelamangala, Bangalore, India; University of Debrecen

**Keywords:** ciprofloxacin, infusion, neutropenic, PK/PD, rat, thigh, pharmacodynamics, pharmacokinetics

## Abstract

The neutropenic mouse infection model is extensively used to characterize the pharmacokinetics/pharmacodynamics (PK/PD) of anti-infective agents. However, it is difficult to evaluate agents following intravenous (i.v.) infusions using this model. Furthermore, in many drug discovery programs, lead identification and optimization is performed in rats, and pharmacology is performed in mice. Alternative models of infection are needed for robust predictions of PK/PD in humans. The rat is an alternative model of infection which can overcome the shortcomings of the mouse model. However, the rat neutropenic thigh infection (NTI) model has not been adequately characterized for evaluation of the PK/PD of anti-infectives. The aim of this study was to characterize the PK/PD of ciprofloxacin against bacterial pathogens in a rat NTI model. We studied the PK/PD relationships of ciprofloxacin against wild-type Escherichia coli, Acinetobacter baumannii, Pseudomonas aeruginosa, and Klebsiella pneumoniae in neutropenic Wistar rats following administration of 10, 30, and 100 mg/kg as single intravenous boluses and 30- and 60-min infusions. The PK/PD of ciprofloxacin against all four pathogens was AUC/MIC dependent and independent of the duration of administration at 10, 30, and 100 mg/kg. At human-equivalent rat doses, the PK/PD targets of ciprofloxacin achieved in rats for microbiological cure were similar to those reported in human patients. The neutropenic rat thigh infection model can be used to evaluate anti-infective agents intended to be administered as infusions in the clinic, and it complements the mouse model, increasing the robustness of PK/PD predictions in humans.

**IMPORTANCE** Many antibiotics are administered as intravenous infusions in the clinic, especially in intensive care units. Anti-infective drug discovery companies develop clinical candidates that are intended to be administered as i.v. infusions in the clinic. However, there are no well-characterized models with which they can evaluate the PK/PD of the candidates following i.v. infusions. The neutropenic rat thigh infection model reported in this study helps in evaluating anti-infective agents that are intended to be administered as i.v. infusions in the clinic. The rat model is useful for simulating the clinical conditions for i.v. infusions for treatment of infections, such as acute bacterial skin and skin structure, lung, and urinary tract infections. This model is predictive of efficacy in humans and can serve as an additional confirmatory model, along with the mouse model, for determining the proof of concept and for making robust predictions of efficacy in humans.

## INTRODUCTION

The neutropenic mouse thigh infection model is considered to be the gold standard for evaluating the efficacy of anti-infective agents because of its high degree of translation to human patients, and it is therefore used to set pharmacokinetic/pharmacodynamic (PK/PD) targets in the clinic to achieve a clinical and microbiological cure (1–9). However, the mouse model is not suitable for (i) evaluation of the pharmacokinetics/pharmacodynamics of anti-infective agents following intravenous (i.v.) infusions or (ii) drug discovery programs in which the drug metabolism and pharmacokinetics (DMPK) of the lead compounds are optimized using PK data from rats, and pharmacology (efficacy) studies are performed in mice. It has also been reported that predictions of anti-infective efficacy made using mouse models have not always translated to humans (9). Alternative infection models are needed to add confidence in making predictions of efficacy for anti-infective agents in humans (10).

The rat is a suitable alternative model for infection studies and has been used for evaluation of the pharmacodynamic activity of anti-infective agents in thigh infection (11, 12), groin abscess (13–15), lung infection (16, 17), and endocarditis (18).

The neutropenic rat thigh infection model is the equivalent of the mouse thigh infection model, which mimics soft tissue infections (11). However, unlike the mouse model, this model has not been used routinely for evaluating anti-infective agents because of its insufficient characterization of the PK/PD of anti-infectives in both the oral and intravenous settings (12). The aim of this investigation was to evaluate the neutropenic rat thigh infection model for characterizing the PK/PD of ciprofloxacin following i.v. infusions against Gram-negative pathogenic bacteria.

(Part of this work was presented at the 29th European Congress of Clinical Microbiology and Infectious Diseases [ECCMID], Amsterdam, Netherlands [19]).

## RESULTS

Characterization of the neutropenic thigh infection model in rats for PK/PD was performed using ciprofloxacin against four different Gram-negative, clinically important bacterial pathogens in three steps: (i) simultaneous PK/PD against Escherichia coli and Pseudomonas aeruginosa following single i.v. bolus doses; (i) efficacy against E. coli and P. aeruginosa following single i.v. infusions; (iii) efficacy against E. coli, P. aeruginosa, A. baumannii, and Klebsiella pneumoniae at human-equivalent rat doses following i.v. infusions.

Ciprofloxacin showed potent inhibition of all four bacterial species, with MICs of 0.03125, 0.125, 0.5, and 0.25 μg/mL against Escherichia coli, Pseudomonas aeruginosa, Acinetobacter baumannii, and Klebsiella pneumoniae, respectively (Table 1).

**TABLE 1:** MICs of ciprofloxacin against different bacteria estimated using the *in vitro* microtiter plate broth dilution method

Strain	MIC (μg/mL)
Escherichia coli ATCC 25922	0.03125
Pseudomonas aeruginosa ATCC 27853	0.125
Acinetobacter baumannii ATCC 19606	0.5
Klebsiella pneumoniae ATCC 13883	0.25

**FIG 1:**
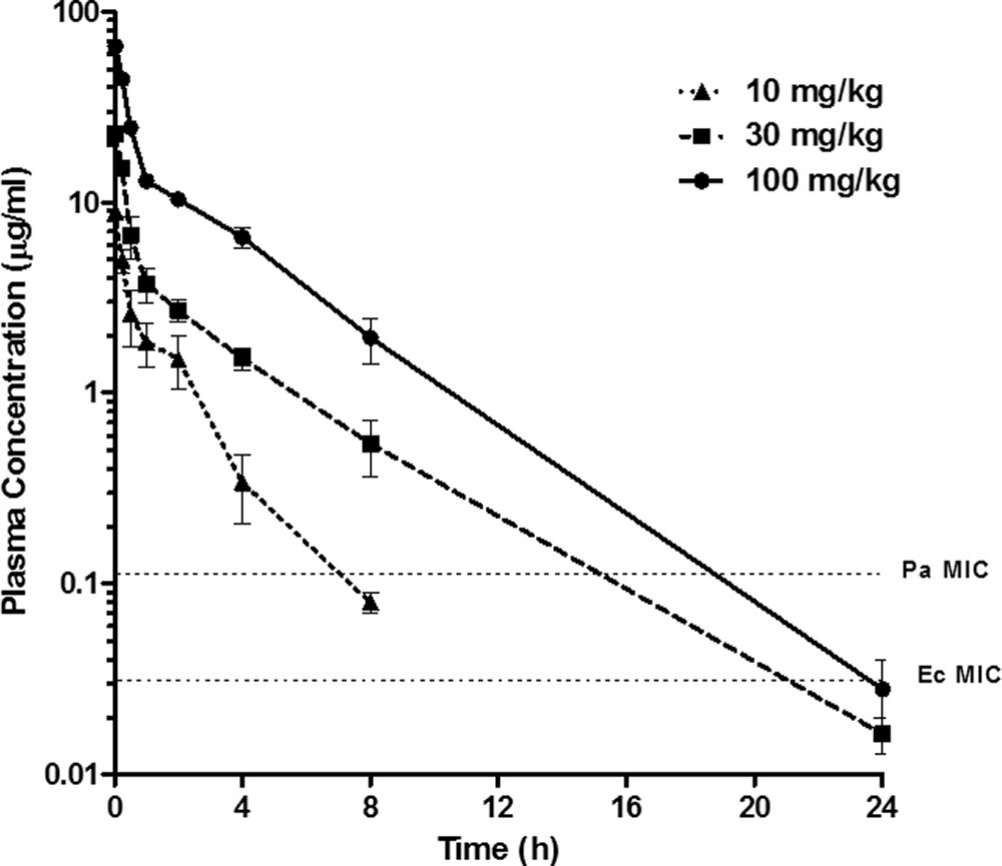
Mean plasma concentration-time profiles of ciprofloxacin in neutropenic infected rats following single i.v. bolus doses of 10 mg/kg (triangles), 30 mg/kg (squares), and 100 mg/kg (circles). Error bars represent the SEM. The dashed horizontal lines represent the MIC concentrations of ciprofloxacin against Ec (Escherichia coli [ATCC 25922]) and Pa (Pseudomonas aeruginosa [ATCC 27853]).

The PK profiles of ciprofloxacin following single i.v. bolus administrations in neutropenic rats infected with E. coli and P. aeruginosa are shown in Fig. 1, and the PK parameters and PK/PD ratios are summarized in Table 2. Ciprofloxacin showed biexponential disposition with first-order kinetics (Fig. 1) characterized by a high volume of distribution at steady state (*V*_ss_; mean *V*_ss_, 2.3 to 4 L/kg), moderate systemic clearance (CL; mean CL, 1.2 to 1.3 L/kg), and moderate elimination half-life (*t*_1/2_; mean *t*_1/2_, 1.5 to 3 h). Linear PK was observed, with a dose-proportional increase in the maximum concentration of drug in serum (*C*_max_) and area under the concentration-time curve from 0 h to infinity (AUC_0–∞_) between 10 and 100 mg/kg (Table 2).

**TABLE 2:** Pharmacokinetic parameters and PK/PD ratios of ciprofloxacin in neutropenic rats infected with E. coli ATCC 25922 and P. aeruginosa ATCC 27853 following single i.v. bolus administrations

PK parameter	Mean ± SD for dose (mg/kg):^*a*^		
	10	30	100
*C*_max_ (μg/mL)	8.95 ± 0.74	22.8 ± 2.0	65.43 ± 4.5
AUC_0–∞_ (μg·h/mL)	7.78 ± 2.14	23.3 ± 5.1	81.52 ± 2.41
*t*_1/2_ (h)	1.54 ± 0.18	3.0 ± 0.29	2.48 ± 0.5
*V*_ss_ (L/kg)	2.30 ± 0.74	3.95 ± 0.5	3.64 ± 0.33
CL (L/h/kg)	1.35 ± 0.33	1.33 ± 0.3	1.23 ± 0.04
PK/PD ratios^*b*^			
*f*AUC_24_/MIC_Ec_	174	522	1,826
*f*AUC_24_/MIC_Pa_	44	130	457
*fC*_max_/MIC_Ec_	200	511	1,466
*fC*_max_/MIC_Pa_	50	128	366

a *n* = 3.

b PK/PD ratios were estimated using the unbound concentrations of ciprofloxacin in rat plasma using *f*_u_ = 0.7 (see Materials and Methods). MIC_Ec_, MIC against E. coli (ATCC 25922); MIC_Pa_, MIC against P. aeruginosa (ATCC 27853).

The mean bacterial load in rat thighs, at 2 h postinfection, was 6.12 log_10_ CFU/g thigh for E. coli (Table 3) and 6.13 log_10_ CFU/g thigh for P. aeruginosa (Table 4).

**TABLE 3:** Pharmacodynamics of ciprofloxacin in neutropenic rats infected with Escherichia coli ATCC 25922 following single i.v. administrations as bolus and infusions^*a*^

Treatment	Mean ± SD E. coli burden (log_10_ CFU/g thigh [reduction^*b*^])		
	i.v. bolus	30-min i.v. infusion	1-h i.v. infusion
Infection control (2 h PI)	6.12 ± 0.41	NA	NA
Vehicle control (26 h PI)	8.76 ± 0.71	NA	NA
Ciprofloxacin, 26 h PI^*c*^ (mg/kg)			
10	4.56 ± 0.23^*d*^ (1.6)	4.02 ± 0.19^*d*^^,^^*e*^ (2.1)	3.72 ± 0.10^*d*^^,^^*e*^ (2.4)
30	4.12 ± 0.27^*d*^ (2.0)	3.48 ± 0.33^*d*^^,^^*f*^ (2.6)	3.25 ± 0.02^*d*^^,^^*f*^ (2.9)
100	1.77 ± 0.04^*d*^ (4.4)	BLOQ	BLOQ

a BLOQ, below the limit of quantitation; NA, not applicable; PI, postinfection.

b Values in parentheses indicate the mean reduction in bacterial burden compared to that of the 2-h PI control at 24 h posttreatment.

c 26 h PI is equivalent to 24 h posttreatment.

d Significantly different compared to 2-h PI control (*P* < 0.05).

e Not significantly different compared to 10 mg/kg i.v. bolus (*P* > 0.05).

f Not significantly different compared to 30 mg/kg i.v. bolus (*P* > 0.05).

**TABLE 4:** Pharmacodynamics of ciprofloxacin in neutropenic rats infected with Pseudomonas aeruginosa ATCC 27853 following single i.v. administrations as bolus and infusions^*a*^

Treatment	Mean ± SD P. aeruginosa burden (log_10_ CFU/g thigh [reduction^*b*^])		
	i.v. bolus	30-min i.v. infusion	1-h i.v. infusion
Infection control (2 h PI)	6.13 ± 0.22	NA	NA
Vehicle control (26 h PI)	8.47 ± 0.13	NA	NA
Ciprofloxacin, 26 h PI^*c*^ (mg/kg)			
10	6.5 ± 0.11^*d*^	6.6 ± 0.12 ^*d*^^,^^*e*^	7.66 ± 0.2^*f*^^,^^*g*^
30	5.3 ± 0.28^*h*^ (0.83)	5.2 ± 0.08^*h*^^,^^*i*^ (0.93)	4.2 ± 0.2^*h*^^,^^*j*^ (1.9)
100	2.2 ± 0.18^*h*^ (3.9)	1.86^*k*^ (4.3)	BLOQ

a NA, not applicable; PI, post infection; BLOQ, below the limit of quantitation.

b Values in parentheses indicate the mean reduction in bacterial burden compared to that of the 2-h PI control at 24 h posttreatment.

c 26 h PI is equivalent to 24 h posttreatment.

d Not significantly different compared to 2-h PI control (*P* < 0.05).

e Not significantly different compared to 10 mg/kg i.v. bolus (*P* > 0.05).

f Significantly different compared to 10 mg/kg i.v. bolus (*P* < 0.05).

g Significantly different from vehicle control (*P* < 0.05).

h Significantly different compared to 2-h PI control (*P* < 0.05).

i Not significantly different compared to 30 mg/kg i.v. bolus (*P* > 0.05).

j Significantly different compared to 30 mg/kg i.v. bolus (*P* < 0.05).

k Mean (*n* = 2).

A ratio of the area under the concentration-time curve for the free, unbound fraction of a drug (*f*AUC) to MIC of ≥62 to 75 is predictive of a clinical and microbiological cure for fluoroquinolones, such as ciprofloxacin, against infections caused by Gram-negative pathogens in the mouse thigh infection model and in the clinic (3). The *f*AUC at 24 h (*f*AUC_24_)/MIC ratios for ciprofloxacin were 174, 522, and 1,826 for E. coli and 44, 130, and 457 for P. aeruginosa in neutropenic rats following single i.v. bolus doses (Table 2). These values were above 75 (except for P. aeruginosa at 10 mg/kg) and thus predicted efficacy in the model. Consistent with these predictions, ciprofloxacin showed a significant dose-dependent antibacterial effect against E. coli compared to the vehicle control (*P* < 0.05). The mean bacterial loads were 4.56, 4.12, and 1.77 log_10_ CFU/g thigh at 10, 30, and 100 mg/kg, respectively, whereas those for the vehicle control and the 2-h control were 8.76 and 6.12 log_10_ CFU/g thigh (Table 3). A significant antibacterial effect was observed against P. aeruginosa following single i.v. bolus doses compared to the vehicle control (*P* < 0.05). The mean bacterial loads were 6.5, 5.3, and 2.2 log_10_ CFU/g thigh at 10, 30, and 100 mg/kg, respectively; the mean bacterial load for the vehicle control was 8.47 log_10_ CFU/g thigh, and that for the 2-h control was 6.13 log_10_ CFU/g thigh (Table 4). The efficacy was not significant compared to the 2-h control at 10 mg/kg against P. aeruginosa (Table 4).

The efficacy of ciprofloxacin in mice and humans is best described by the PK/PD index AUC/MIC (1, 3, 7). We reasoned that the AUC/MIC should also be the driver for efficacy in rats. Normally, dose fractionation studies are performed to identify the PK/PD index in mice, where the same total dose is divided and administered as equally divided doses once, twice, or thrice in 24 h (1, 3). In this study, three dose levels (10, 30, and 100 mg/kg) of ciprofloxacin were administered to infected rats as an i.v. bolus or 30- or 60-min infusions in 24 h. Thus, assuming linear PK, for each total dose, the AUC/MIC should be the same, whereas the *C*_max_/MIC and the time that the drug concentration exceeds the MIC under steady-state pharmacokinetic conditions (*T*_>MIC_) will vary (4, 8). The efficacy of ciprofloxacin when administered as 30-min and 1-h i.v. infusions was similar to that of the i.v. bolus at each of the dose levels (10, 30, and 100 mg/kg) (Table 3). The mean E. coli burdens for the i.v. bolus and 30- and 60-min infusions at 10 mg/kg were 4.56, 4.02, and 3.72 log_10_ CFU/g thigh, respectively; at 30 mg/kg, they were 4.12, 3.48, and 3.25 log_10_ CFU/g thigh, and at 100 mg/kg, they were 1.77 log_10_ CFU/g thigh, below the level of quantitation (BLOQ), and BLOQ, respectively (Table 3).

Similar results were observed for P. aeruginosa (Table 4). The P. aeruginosa bacterial loads for the i.v. bolus and 30- and 60-min infusions were 6.5, 6.6, and 7.7 log_10_ CFU/g thigh at 10 mg/kg; 5.3, 5.2, and 4.2 log_10_ CFU/g thigh at 30 mg/kg; and 2.2 and 1.86 log_10_ CFU/g thigh and BLOQ at 100 mg/kg, respectively (Table 4).

**FIG 2:**
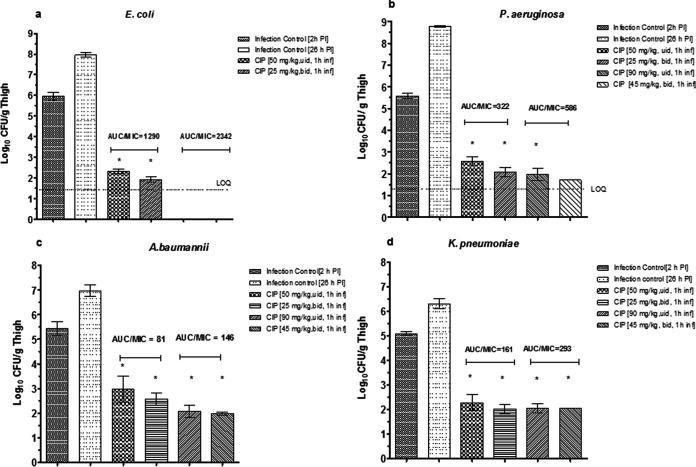
Pharmacodynamics of ciprofloxacin against Escherichia coli ATCC 25922 (a), Pseudomonas aeruginosa ATCC 27853 (b), Acinetobacter baumannii ATCC 19606 (c), and Klebsiella pneumoniae ATCC 13883 (d) at human-equivalent rat doses in the rat neutropenic thigh infection model following i.v. infusions (see Materials and Methods for details). The AUC/MIC ratio for each treatment is indicated above the corresponding bar. Error bars represent the mean and standard deviation. Asterisks indicate significant differences compared to the 2-h infection control (*P* < 0.05). LOQ, limit of quantitation.

Ciprofloxacin is administered at 200 mg or 400 mg, twice daily, as 1-h i.v. infusions, to treat critically ill patients (7, 20). We simulated this situation in rats infected with four different Gram-negative bacterial pathogens, at rat equivalents of the human doses. The results are shown in Fig. 2. Ciprofloxacin showed significant efficacy (*P* < 0.05) compared to the baseline bacterial load in thighs, with >2 log_10_ CFU/g thigh killing of E. coli (ATCC 25922), P. aeruginosa (ATCC 27853), A. baumannii (ATCC 19606), and K. pneumoniae (ATCC 13883) cells at both the human-equivalent rat doses of 45 mg/kg and 90 mg/kg. This finding was consistent with the ratio of total AUC/MIC of >125 in rats for all four pathogens (Fig. 2).

It was observed that for rat doses of 45 mg/kg (equivalent to a human dose of 400 mg) and 90 mg/kg (equivalent to a human dose of 800 mg), the efficacies were similar when the doses were given as a single dose or two divided doses (Fig. 2), suggesting that the AUC/MIC was the driver of efficacy for ciprofloxacin against all four pathogens.

A comparison of the PK/PD ratios and efficacy in neutropenic rats and humans is shown in Table 5. The total *C*_max_/MIC ratios achieved in the neutropenic rat thigh infection model were 69 to 1,102 and 38 to 604 at rat doses of 50 mg/kg and 119 to 1,900 and 62 to 992 at 90 mg/kg for the once-daily and twice-daily regimens, respectively (Table 5); the ratios were found to be within the range observed in these patients (6.2 to 5,541) (7). Similarly, the range for *C*_max_, a parameter associated with emergence of resistance, was 19 to 59 in the neutropenic rats (Table 5), which was in the range observed in human patients (0.9 to 769) (Table 5).

**TABLE 5:** Comparison of pharmacokinetics/pharmacodynamics and efficacy of ciprofloxacin in the neutropenic rat thigh infection model with data from humans

Species	Total dose (regimen)^*a*^	Value (range)				Efficacy (% microbiological cure)
		AUC_24_ (μg·h/mL)	AUC_24_/MIC	*C*_max_ (μg/mL)	*C*_max_/MIC	
Humans^*b*^	400 mg (7 mg/kg − 200 mg, b.i.d., i.v.)	49 (9.0–229)	802 (6.2–5,541)	4.8 (1.9–15.4)	100 (0.9–769)	88% clinical cure (81)
Rat (human-equivalent dose)^*c*^	50 mg/kg (u.i.d.)^*d*^	40	81–1,290	34	69–1,102	≥3 log_10_ CFU/g thigh reduction (99)
	50 mg/kg (25 mg/kg, b.i.d.)^*d*^	40	81–1,290	19	38–604	≥3 log_10_ CFU/g thigh reduction (99)
	90 mg/kg (u.i.d.)^*e*^	73	146–2,342	59	119–1,900	≥3 log_10_ CFU/g thigh reduction (99)
	90 mg/kg (45 mg/kg, b.i.d.)^*e*^	73	146–2,342	31	62–992	≥3 log_10_ CFU/g thigh reduction (99)

a b.i.d., twice a day; u.i.d., once a day.

b Human data are from reference 7.

c See Materials and Methods for calculations.

d Rat equivalent of a human total dose of 400 mg (7).

e Rat equivalent of a human total dose of 800 mg (21).

## DISCUSSION

The PK/PD of ciprofloxacin were evaluated in a neutropenic thigh infection model in rats. The mean bacterial load in rat thighs, at 2 h postinfection, was 6.12 log_10_ CFU/g thigh for E. coli and 6.13 log_10_ CFU/g thigh for P. aeruginosa. These values were comparable to the bacterial loads in thighs of Gram-negative bacilli observed in the mouse neutropenic thigh infection model at 2 h postinfection (21).

The efficacy of ciprofloxacin against E. coli and P. aeruginosa following single-dose i.v. administration was consistent with the *f*AUC/MIC ratios exceeding 75, which was comparable to that in mice and humans (3).

The efficacy of ciprofloxacin against E. coli and P. aeruginosa was comparable between i.v. bolus and infusion administrations for each dose level. This result could be explained by the PK principle that the AUC of a drug is the same whether it is administered as an i.v. bolus or infusion, but the *C*_max_ will vary (22). However, this explanation needs to be verified by performing PK studies in animals in the infusion groups and comparing the results with those of the i.v. bolus group. It follows that all three regimens for a given dose yielded the same *f*AUC/MIC and thus showed similar efficacies. The method described above can be used to perform i.v. dose fractionation studies in rats to identify the PK/PD indices of efficacy for anti-infective agents intended to be administered as i.v. infusions in the clinic. To our knowledge, this is the first report of dose fractionation studies performed using i.v. administration regimens in a rodent model of infection.

At human-equivalent doses in rats, ciprofloxacin achieved therapeutic exposure (AUC/MIC and *C*_max_/MIC) comparable to that achieved in humans with different infections (7). In the study by Forrest et al. (7), ciprofloxacin showed a >80% clinical and microbiological cure in patients (with soft tissue and urinary tract infections and bacteremia) when the ratio of total AUC/MIC was ≥125 and remained similar when the AUC/MIC was >250 and up to 5,000. Importantly, the infections observed in the patients included Pseudomonas and other Gram-negative bacteria which were used in our model. In another study in which ciprofloxacin was administered (400 mg twice daily) to patients with infections of the skin and skin structure, lower respiratory tract, and urinary tract, a 93% clinical response was observed (23). In our study, when the same rat equivalent of a human dose was administered, a 99.9% microbiological cure was observed.

The immunocompetent rat groin abscess model has been used to study the PK/PD of anti-infective agents (13) and to evaluate human-equivalent doses as infusions (14); the immunocompetent rat lung infection model has been used to evaluate human-equivalent i.v. infusion doses (24). However, these models are not the equivalent of the neutropenic mouse infection models (1, 3). Although the rat neutropenic thigh infection model was used to evaluate the efficacy of vancomycin using imaging (11), this model was not subsequently used for characterization of the PK/PD of anti-infective agents. To our knowledge, this is the first report of the characterization of i.v. infusion PK/PD of an antibiotic in the neutropenic rat thigh infection model.

To strengthen and further validate this model, the PK/PD relationships (dose-response relationships, identification of the PK/PD index for efficacy) of other classes of anti-infective drugs should be characterized using different clinically important bacterial pathogens and compared with those of the neutropenic mouse thigh infection PK/PD model.

The rat neutropenic thigh infection model can be used (i) to evaluate the efficacy of anti-infective agents intended to be used for i.v. infusion therapy; (ii) in situations where rats are the model for PK and mice are used for PD studies; (iii) to simulate clinical dosing scenarios; and (iv) as an alternate model for confirmation of the efficacy observed in mice to enable more robust predictions of the efficacy in humans.

## MATERIALS AND METHODS

### Bacterial strains.

Escherichia coli ATCC 25922, Acinetobacter baumannii ATCC 19606, Pseudomonas aeruginosa ATCC 27853, and Klebsiella pneumoniae ATCC 13883 were acquired from the American Type Culture Collection.

### Reagents and chemicals.

Ciprofloxacin (CIP) (Sigma-Aldrich, India; catalog no. 127M4065V), cyclophosphamide (TCI Chemicals; catalog no. C2236), EDTA (Fisher Scientific, India; lot no. 2251370614), ketamine hydrochloride (Neon Laboratories, India; batch no. 982031), xylazine (Indian Immunologicals, India; batch no. FHK8003), and casein soybean digest agar (CSDA) (catalog no. MH011), casein soybean digest broth (CSDB) (catalog no. M290), MacConkey broth (catalog no. MH083), and MacConkey agar (catalog no. GM082) from HiMedia (India) were used in the studies. All other reagents were of analytical grade.

### MICs and inoculum for infection.

**(i) MICs.** The MIC assays were performed based on CLSI guidelines in a microtiter plate format (25) in a total assay volume of 200 μL.

**(ii) Inoculum for infection.** Bacterial colonies for each bacterial species were picked from isolated colonies and grown in sterile CSDB broth (Escherichia coli ATCC 25922, Acinetobacter baumannii ATCC 19606, Pseudomonas aeruginosa ATCC 27853) or MacConkey broth (Klebsiella pneumoniae ATCC 13883) at 37°C. On the day of infection, the overnight culture was centrifuged at 3,000 rpm for 15 min; the cells were then harvested, washed with phosphate-buffered saline (PBS; pH 7.4), and resuspended in sterile buffer. The optical density at 600 nm of the suspension was adjusted to ~1.0 (~1 × 10^7^ CFU/mL) and used for infections. The inoculum used for infection was serially diluted in sterile broth, and 0.05 mL of each dilution was plated onto sterile preincubated CSDA or MacConkey agar plates for enumeration.

### Animal studies.

The animal studies were conducted based on protocols approved by the Institutional Animal Ethics Committee (affiliated with the Committee for the Purpose of Control and Supervision of Experiments on Animals, Ministry of Animal Welfare, Government of India). Male Wistar rats (Vivo Biotech, Hyderabad, India), aged 6 to 8 weeks, were used in all the studies. The animals were housed in individually ventilated cage systems and provided with daily cycles of 12 h light and 12 h darkness. Feed (SDS Diets, India) and autoclaved drinking water were provided *ad libitum*. The animal room temperature range was 22°C ± 3°C, and the humidity range was 30% to 70%.

### Neutropenic thigh infection model in rats.

Four days before the day of infection, each rat was dosed with a single intraperitoneal injection of cyclophosphamide equivalent to 150 mg/kg and returned to its cage. One day before infection, each rat received a dose equivalent to 100 mg/kg of cyclophosphamide. This procedure was based on previous reports for mice (1, 4) and rats (11) and is designed to ensure that the animals will be neutropenic on the day of infection. This was further confirmed by the white blood cell counts in blood samples drawn on the day of infection (mean ± SD neutrophils, 2.4% ± 1.4%). One day before infection (day −1), single colonies of bacteria, obtained by plating from glycerol stocks of Escherichia coli ATCC 25922, Acinetobacter baumannii ATCC 19606, Pseudomonas aeruginosa ATCC 27853, and Klebsiella pneumoniae ATCC 13883, were inoculated into the appropriate sterile broth media mentioned previously and incubated overnight at 37°C. On the day of infection, the overnight cultures were centrifuged; the cell pellets were resuspended in sterile normal saline, serially diluted to obtain approximately 1 × 10^7^ CFU/mL, and used for infection. The inocula were serially diluted 10-fold in sterile broth, and 0.05 mL of six dilutions for each species were plated onto the appropriate agar plates to determine the viable count of inoculum. The infections were conducted in a biological safety cabinet, with appropriate personal protection. Infection was performed by injecting 0.2 mL of inoculum of each bacterial species (~1 × 10^7^ CFU/mL in sterile saline) into each thigh (the right thigh was injected with Escherichia coli ATCC 25922 or Acinetobacter baumannii ATCC 19606; the left thigh was injected with 0.2 mL of Pseudomonas aeruginosa ATCC 27853 or Klebsiella pneumoniae ATCC 13883), using a 1.0-mL syringe and needle. Each rat was infected with approximately 2 × 10^6^ CFU bacterial species per thigh.

### Single-dose intravenous pharmacokinetic/pharmacodynamic studies.

Neutropenic rats (*n* = 3 per group) were infected with (approximately 2 × 10^6^ CFU per thigh) Pseudomonas aeruginosa ATCC 27853 in the left thigh and Escherichia coli ATCC 25922 in the right thigh. At 2 h postinfection, the animals were treated intravenously with the vehicle and ciprofloxacin, either as bolus doses or as constant rate infusions (6-channel syringe infusion pump; Yashtech, India) (0.03 mL/min) over 30 min or 1 h, under anesthesia (ketamine 60 mg/kg intraperitoneally [i.p.] plus xylazine 10 mg/kg i.p.) at a dose volume of 10 mL/kg. The duration of the treatment was 24 h. Ciprofloxacin was formulated in MilliQ water (pH 4.5). At the end of treatment, the animals were sacrificed with an overdose of carbon dioxide and dipped into 70% ethanol for surface decontamination. The thigh muscles were aseptically excised, weighed, placed into 2 mL of sterile saline, and homogenized (Omni Tip handheld tissue homogenizer; THB220). Serial 10-fold dilutions of the thigh homogenates were prepared in sterile saline, and 0.05 mL of four dilutions for each thigh was plated onto CSDA agar plates and incubated at 37°C for 24 h. Bacterial densities were estimated as log_10_ CFU/g of thigh.

**(i) Pharmacokinetics.** The PK was evaluated for ciprofloxacin in the same groups of infected rats at 10, 30, and 100 mg/kg, in the groups receiving i.v. bolus single doses. Postdose serial blood samples were collected (0.1 mL blood per sample) via the retro-orbital plexus at different time points from each rat, and the terminal sampling was done by cardiac puncture. Blood samples were collected in 1.5-mL Eppendorf tubes containing 5 μL of 10% dipotassium EDTA (K_2_-EDTA), mixed gently, and centrifuged at 10,000 rpm for 2 min; the plasma was harvested and stored at −80°C.

**(ii) Bioanalysis.** The ciprofloxacin in rat plasma was quantified by tandem high-performance liquid chromatography (HPLC)-UV and mass spectrometry. Briefly, 50-μL plasma samples were precipitated with 150 μL acetonitrile and centrifuged, and 7 μL of the supernatant was analyzed. A triple-quadrupole mass spectrometer (API 5000; Applied Biosystems) connected to a high-performance liquid chromatograph (Shimadzu SIL-HTc) was used for the analysis. The samples were separated on a reverse-phase column (Atlantis dC_18_ [4.6 by 50 mm], 3 μ) under gradient conditions. The mobile phases A and B were acetonitrile and 0.2% formic acid in MilliQ water, respectively, with a flow rate of 1 mL/min; the run time was 3.5 min. The mass transition used for the detection of ciprofloxacin was *m/z* 332 (parent) and *m/z* 288 (daughter ion) under electrospray ionization (ESI) positive ion mode. The calibration curve was linear (*r*^2^ > 0.99) and accurate and precise between 4.2 and 1,800 ng/mL, and the lower limit of quantitation was 4.2 ng/mL.

**(iii) PK analysis.** Plasma concentration-time profiles were graphed as semilogarithmic plots to assess the first-order kinetics. The noncompartmental (NCA) PK parameters *k*_e_ (elimination rate constant), *V*_ss_ (volume of distribution at steady state), CL (systemic clearance), *C*_max_ (peak plasma concentration), AUC_last_ (area under the plasma concentration-time curve up to the last nonzero concentration), AUC_0–∞_ (AUC extrapolated to infinity), and *t*_1/2_ (terminal half-life) were estimated using plasma concentration-time data from individual rats using Phoenix WinNonLin v8 (Pharsight, Certara). The AUC was computed using the linear up-log down method. The mean and standard deviation estimates for the above parameters were estimated for each dose level. The *C*_max_/MIC and AUC_24_/MIC ratios were estimated using the corresponding MICs of ciprofloxacin for Escherichia coli (ATCC 25922), Pseudomonas aeruginosa (ATCC 27853), Acinetobacter baumannii (ATCC 19606), and Klebsiella pneumoniae (ATCC 13883).

**(iv) PK/PD.** The PK/PD indices of antibacterial efficacy (3) for the ciprofloxacin *fC*_max_/MIC and *f*AUC_24_/MIC ratios were estimated by dividing the free *C*_max_ and free AUC_24_, obtained at 10, 30, and 100 mg/kg i.v. bolus doses, with the corresponding MIC of ciprofloxacin for each bacterium. A plasma protein binding of 30% to rat plasma for ciprofloxacin (23) was used to estimate the free *C*_max_ and AUC_24_.

**Pharmacodynamics of ciprofloxacin at human-equivalent rat doses.** The pharmacodynamics of ciprofloxacin was evaluated in the neutropenic rat thigh infection model at doses equivalent to the human clinical doses of 200 mg, twice a day (total dose, 400 mg/day), i.v. (7), and 400 mg, twice a day (total dose, 800 mg/day), i.v. (20). To estimate the rat equivalents of the human clinical doses of ciprofloxacin, the human doses were converted to milligrams per kilogram for an average human adult of 70 kg. The human dose (mg/kg) was used to estimate the equivalent dose (mg/kg) for a 250-g rat using the following allometric equation based on body surface area (26):

$$\text{animal dose }\left(\text{mg}/\text{kg}\right)\;=\text{ human total dose }{(\text{mg}/\text{kg})/(\text{ animal weight in kg}/\text{human weight in kg})}^{0.\text{33}}$$

The estimated rat equivalents of the total human doses of 400 mg and 800 mg were 43 mg/kg and 90 mg/kg, respectively. The following doses and regimens were used in the study: (i) 50 mg/kg (the 43 mg/kg was rounded up to 50 mg/kg), single dose, 1 h i.v. infusion; (ii) 25 mg/kg, two doses, every 12 h (q12h), 1 h i.v. infusion; (iii) 90 mg/kg, single dose, 1 h i.v. infusion; and (iv) 45 mg/kg, two doses, q12h, 1 h i.v. infusion. One group of neutropenic rats (*n* = 3 per treatment group) was infected with Pseudomonas aeruginosa (ATCC 27853) in the left thigh and Escherichia coli (ATCC 25922) in the right thigh, and another group (*n* = 3 per treatment group) was infected with Klebsiella pneumoniae (ATCC 13883) (left thigh) and Acinetobacter baumannii (ATCC 19606) (right thigh).

Two hours postinfection, the animals were treated with the vehicle and the doses and regimens mentioned above for a period of 24 h. Following treatment, the bacterial loads in thighs were estimated using the procedure described previously. The AUCs associated with the human-equivalent rat doses were obtained by interpolation from the linear regression model of the AUC versus doses from the PK studies mentioned previously. The AUC/MIC ratio was then computed using the corresponding MIC values for each bacterium.

### Statistical analysis.

A one-way analysis of variance (ANOVA), followed by either a Dunnett’s or Bonferroni’s multiple comparison posttest, was used to analyze the statistical significance of differences between the treatment and vehicle control groups in the pharmacodynamic studies. A *P* value of <0.05 was considered statistically significant.
